# Supplementary material for: Dissecting the coordinated progression of cell states in spatial transcriptomics with CoPro
Source: bioRxiv. 2026 Apr 21:2026.04.17.719309. Preprint. [Version 1] doi: 10.64898/2026.04.17.719309 (PMC13131544; doi:10.64898/2026.04.17.719309)
Supplement: 1 [file NIHPP2026.04.17.719309V1-supplement-1.pdf]

bioRxiv preprint doi: https://doi.org/10.64898/2026.04.17.719309; this version posted April 21, 2026. The copyright holder for this preprint (which was not certified by peer review) is the author/funder, who has granted bioRxiv a license to display the preprint in perpetuity. It is made available under aCC-BY 4.0 International license.

Supplementary Materials:

Supplementary Figures 1-19

Supplementary Table 1: Conceptual comparison of methods for spatial transcriptomic analysis

Supplementary Table 2-4: Benchmark performance of CoPro and comparison methods in simulations (naïve, proportions, and orthogonal)

Supplementary Table 5: CoPro gene weights in healthy colon (Day 0). PCA back-projection and regression-based weights for Epithelial, Fibroblast, and Immune cells (CC1).

Supplementary Table 6: CoPro gene weights in DSS-treated colon (Day 3). PCA back-projection and regression-based weights for Epithelial, Fibroblast, and Immune cells (CC1 and CC2).

Supplementary Table 7: Cell-type-specific genes associated with disease severity in DSS-treated colon (Day 9)

Supplementary Table 8: CoPro gene weights in DSS-recovered colon (Day 21). PCA back-projection and regression-based weights for Epithelial, Fibroblast, and Immune cells (CC1).

Supplementary Table 9: CoPro gene weights in healthy mouse liver

Supplementary Table 10: Zonation-associated endothelial genes in healthy liver, quadratic

Supplementary Table 11: Pathway grouping for the mutant liver (*Ercc1*$^{-/\Delta}$)

Supplementary Table 12: CoPro gene weights in kidney tubular segments. PCA back-projection and regression-based weights.

bioRxiv preprint doi: https://doi.org/10.64898/2026.04.17.719309; this version posted April 21, 2026. The copyright holder for this preprint (which was not certified by peer review) is the author/funder, who has granted bioRxiv a license to display the preprint in perpetuity. It is made available under aCC-BY 4.0 International license.

Supplementary Table 13: CoPro gene weights in kidney vasculature. PCA back-projection and regression-based weights.

bioRxiv preprint doi: https://doi.org/10.64898/2026.04.17.719309; this version posted April 21, 2026. The copyright holder for this preprint (which was not certified by peer review) is the author/funder, who has granted bioRxiv a license to display the preprint in perpetuity. It is made available under aCC-BY 4.0 International license.

Supplementary Fig. 1

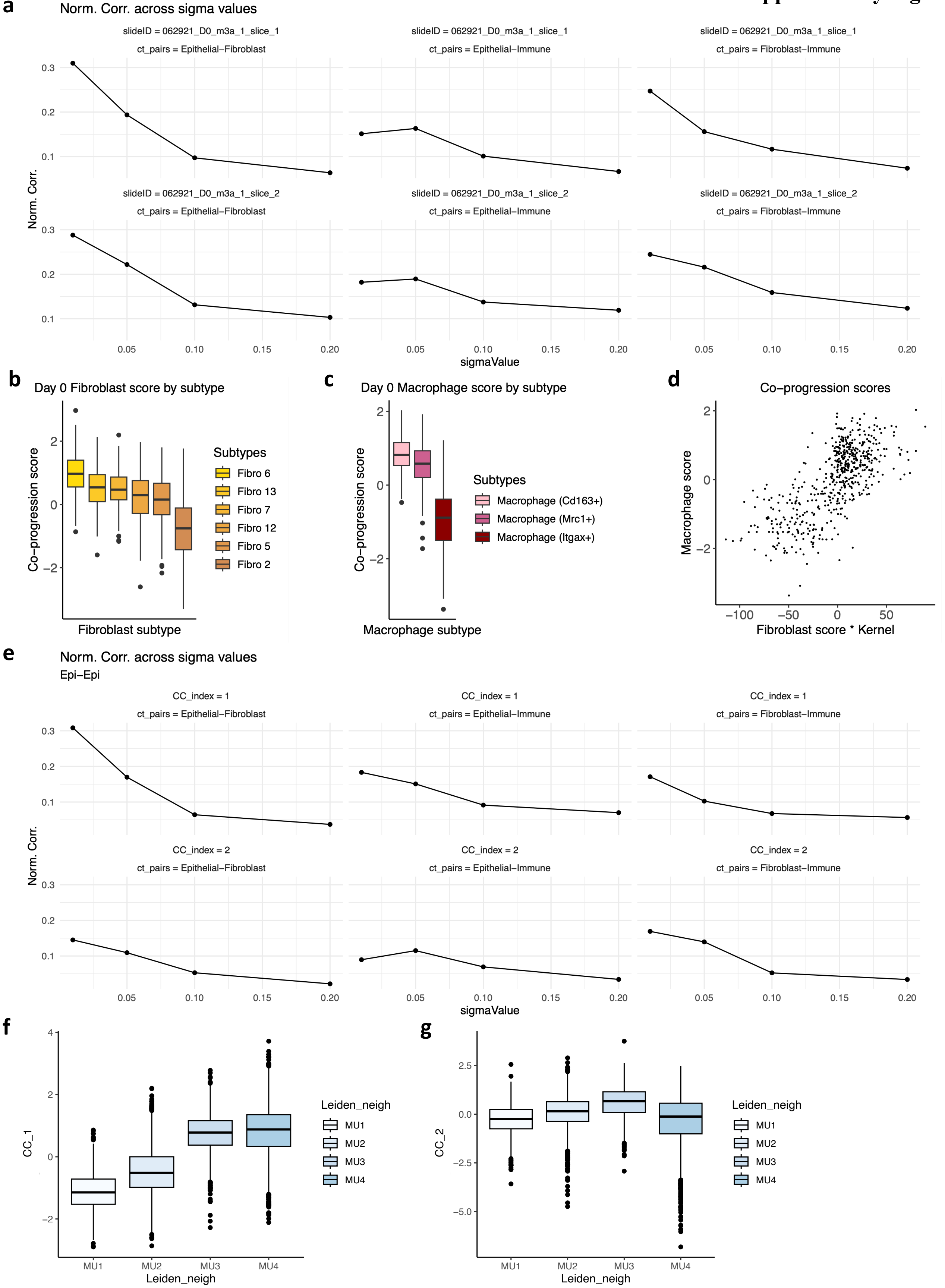

bioRxiv preprint doi: https://doi.org/10.64898/2026.04.17.719309; this version posted April 21, 2026. The copyright holder for this preprint (which was not certified by peer review) is the author/funder, who has granted bioRxiv a license to display the preprint in perpetuity. It is made available under aCC-BY 4.0 International license.

Supplementary Figure 1.

(**a**) Normalized correlation across sigma values for Day 0 colon co-progression analysis, evaluated for all three cell type pairs (Epithelial–Fibroblast, Epithelial–Immune, Fibroblast–Immune) across two representative tissue sections.

(**b**) Fibroblast co-progression scores grouped by subtype at Day 0. The scoring links fibroblast 6 to the crypt base and fibroblast 2 to the crypt tip.

(**c**) Macrophage co-progression scores by subtype at Day 0, revealing that Itgax+ macrophages localize toward the tip while Mrc1+ macrophages localize near the crypt base.

(**d**) Scatter plot of kernel-smoothed co-progression scores between macrophage and fibroblast cell types in a representative Day 0 section

(**e**) Normalized correlation across sigma values for Day 3 colon co-progression analysis, evaluated for all three cell type pairs (Epithelial–Fibroblast, Epithelial–Immune, Fibroblast–Immune) and two retained canonical components (CC1 and CC2).

(**f–g**) Distribution of Day 3 CC1 (**f**) and CC2 (**g**) scores stratified by mucosal microenvironment (ME) neighborhood labels from the original publication.

bioRxiv preprint doi: https://doi.org/10.64898/2026.04.17.719309; this version posted April 21, 2026. The copyright holder for this preprint (which was not certified by peer review) is the author/funder, who has granted bioRxiv a license to display the preprint in perpetuity. It is made available under aCC-BY 4.0 International license.

Supplementary Fig. 2

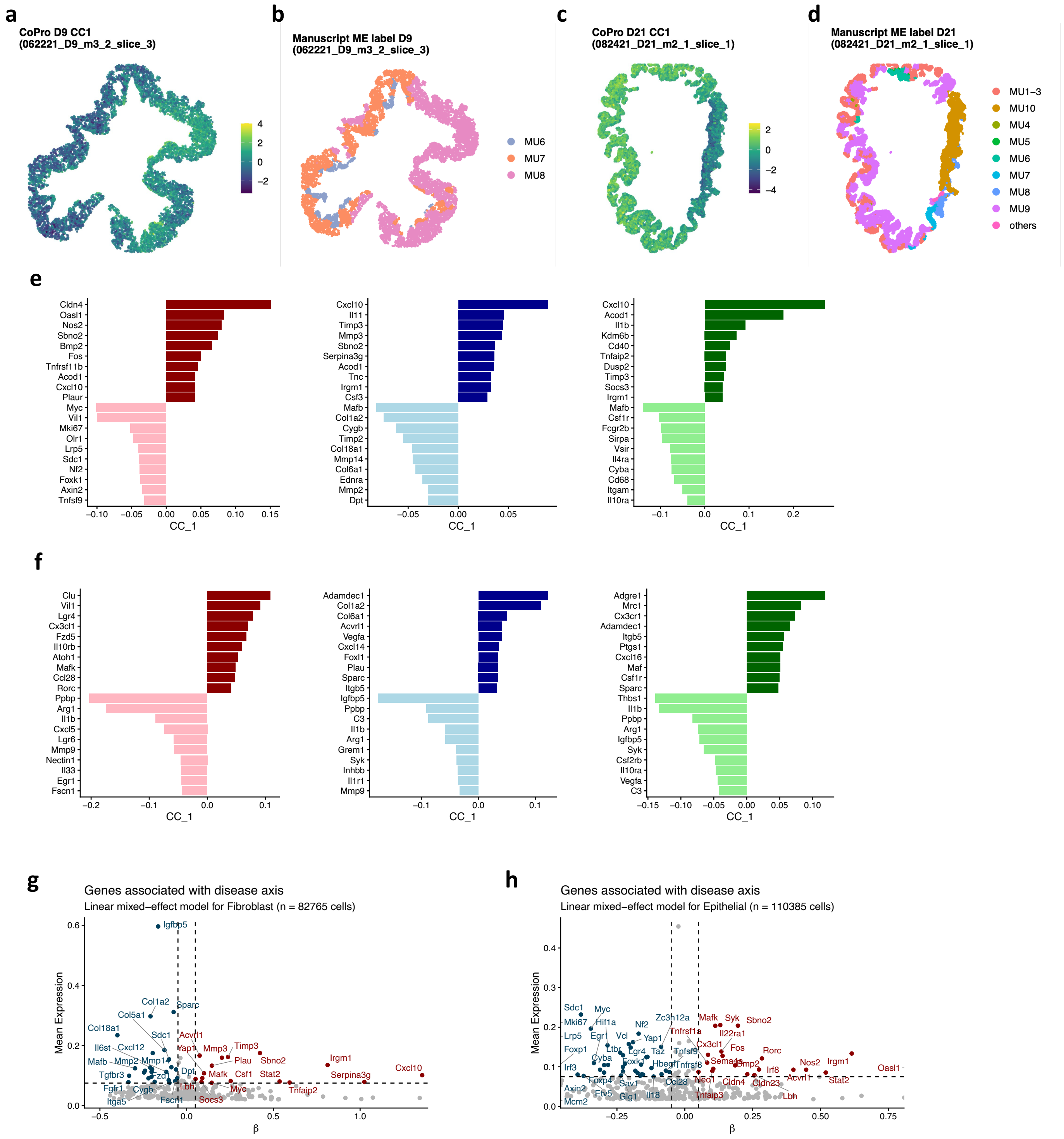

Supplementary Figure 2.

(**a-d**) Spatial maps projecting CoPro canonical component 1 (CC1) scores alongside the manuscript's manually curated microenvironment (ME) labels for Day 9 (severe inflammation, **a-b**) and Day 21 (repair, **c-d**) samples.

(**e-f**) Cell-type-specific gene weights derived from the CoPro progression axes for Epithelial, Fibroblast, and Immune cells at Day 9 (**e**) and Day 21 (**f**).

(**g-h**) Identification of cell-type-specific genes associated with disease severity. Results map the linear mixed-effects model outputs for Fibroblast (**g**) and Epithelial (**h**) cells, adjusting for mouse- and slice-level variation.

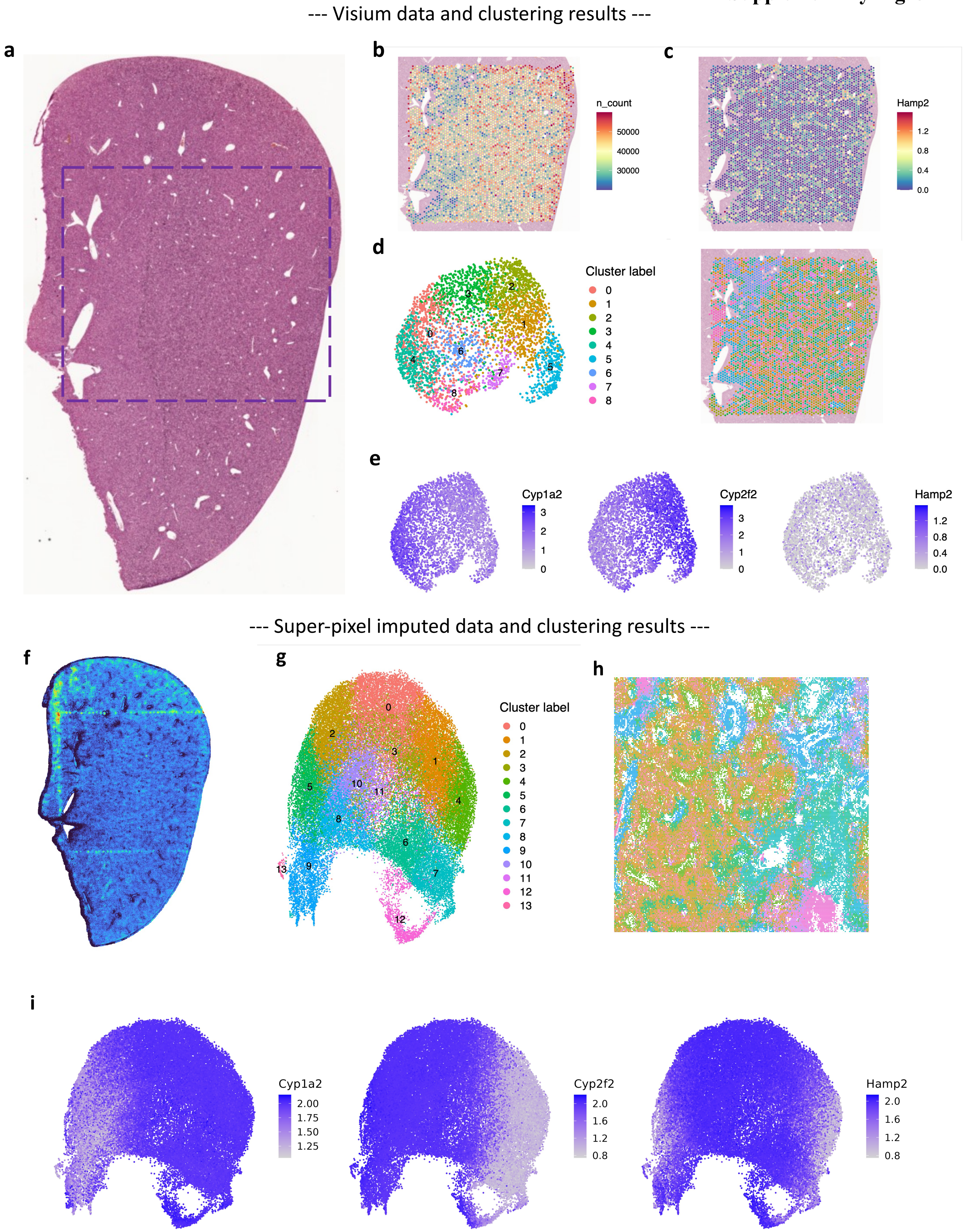
--- Visium data and clustering results ---
a
b
n_count
50000
40000
30000
c
Hamp2
1.2
0.8
0.4
0.0
d
Cluster label
0
1
2
3
4
5
6
7
8
e
Cyp1a2
3
2
1
0
Cyp2f2
3
2
1
0
Hamp2
1.2
0.8
0.4
0.0
--- Super-pixel imputed data and clustering results ---
f
g
Cluster label
0
1
2
3
4
5
6
7
8
9
10
11
12
13
h
i
Cyp1a2
2.00
1.75
1.50
1.25
Cyp2f2
2.0
1.6
1.2
0.8
Hamp2
2.0
1.6
1.2
0.8

Supplementary Figure 3.
(**a**) H&E-stained image of a healthy wild-type mouse liver section. The purple rectangle marks the region captured by Visium experiment.
(**b**) Spatial map of total UMI counts for Visium spots.
(**c**) Spatial expression of a representative zonation marker (*Hamp2*) on Visium spots.
(**d**) Leiden clustering of Visium spots projected onto tissue coordinates.
(**e**) Spatial expression of canonical zonation markers on Visium spots.
(**f**) *Hamp2* expression for the full liver section computed on 8-μm super-pixel imputed data (iSTAR)
(**g-h**) Leiden clustering of iSTAR super-pixels, shown on UMAP (**g**) and tissue coordinates (**h**).
(**i**) Spatial expression of canonical zonation markers on iSTAR super-pixels.

Supplementary Fig. 4

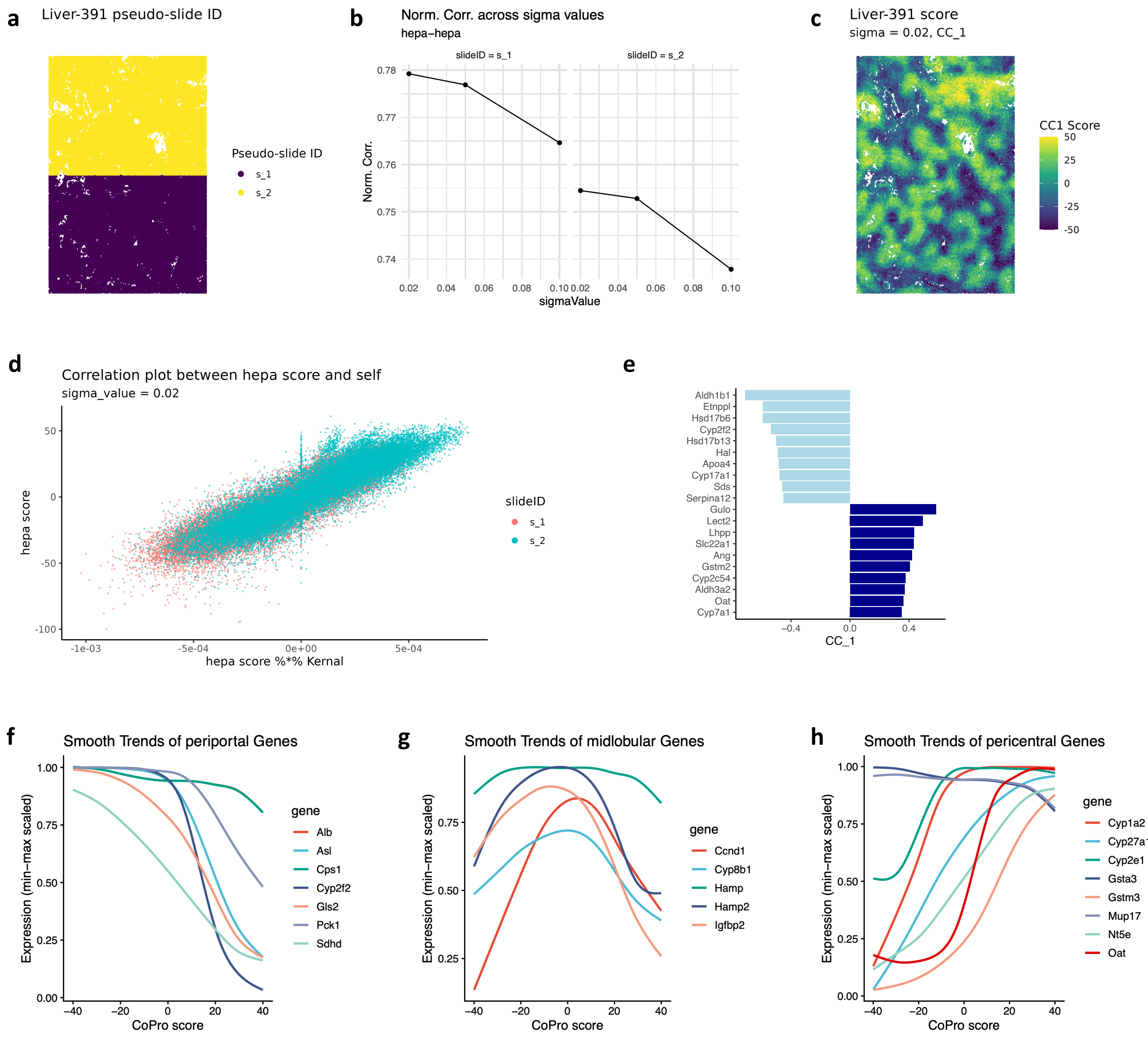

Supplementary Figure 4.

(**a**) Pseudo-slide partition of the Liver-391 training half that enables multi-slide CoPro analysis.

(**b**) Normalized correlation across sigma values for single-cell-type hepatocyte CoPro analysis.

(**c**) CoPro CC1 score map projected onto the Liver-391 section. The spatial pattern shows coherent periportal-to-pericentral organization, with high and low scores localized near vascular structures and intermediate scores spanning the intervening parenchyma.

(**d**) Scatter plot of kernel-smoothed hepatocyte CC1 scores against the cell score, with each point colored by pseudo-slide identity (s_A vs s_B).

(**e**) CC1 gene weight bar plot for hepatocytes.

(**f-h**) Smooth expression trends of canonical zonation markers (f: periportal, g: midlobular, h: pericentral) as a function of CoPro score.

Supplementary Fig. 5

a b

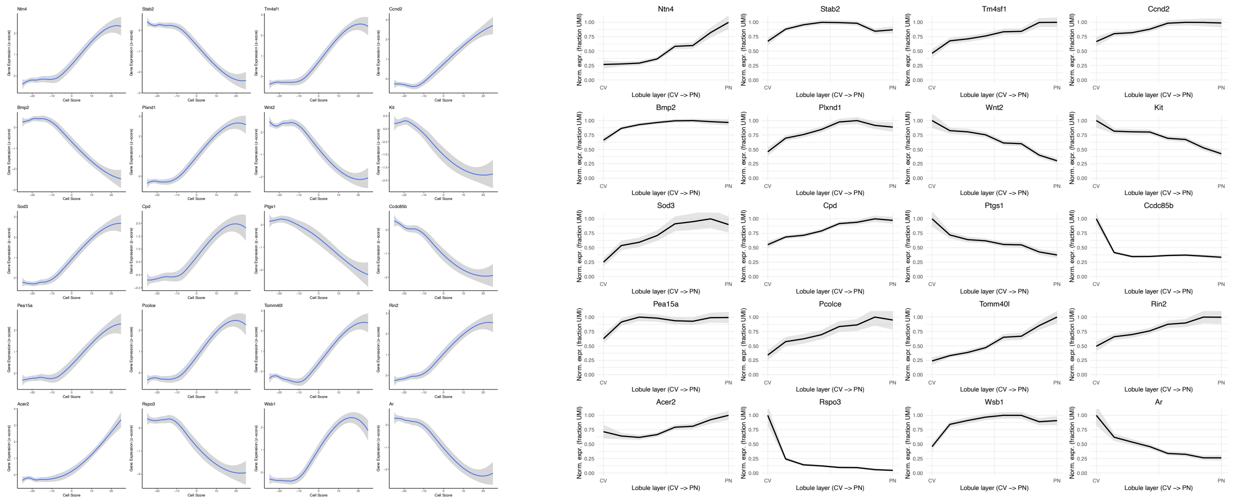

Supplementary Figure 5.

(**a**) Smooth trends of individual endothelial gene expression plotted along the CoPro-inferred zonation score axis.

(**b**) Normalized zonal mean expression for the same genes from published paired-cell RNA sequencing (pcRNA-seq) data across lobular layers.

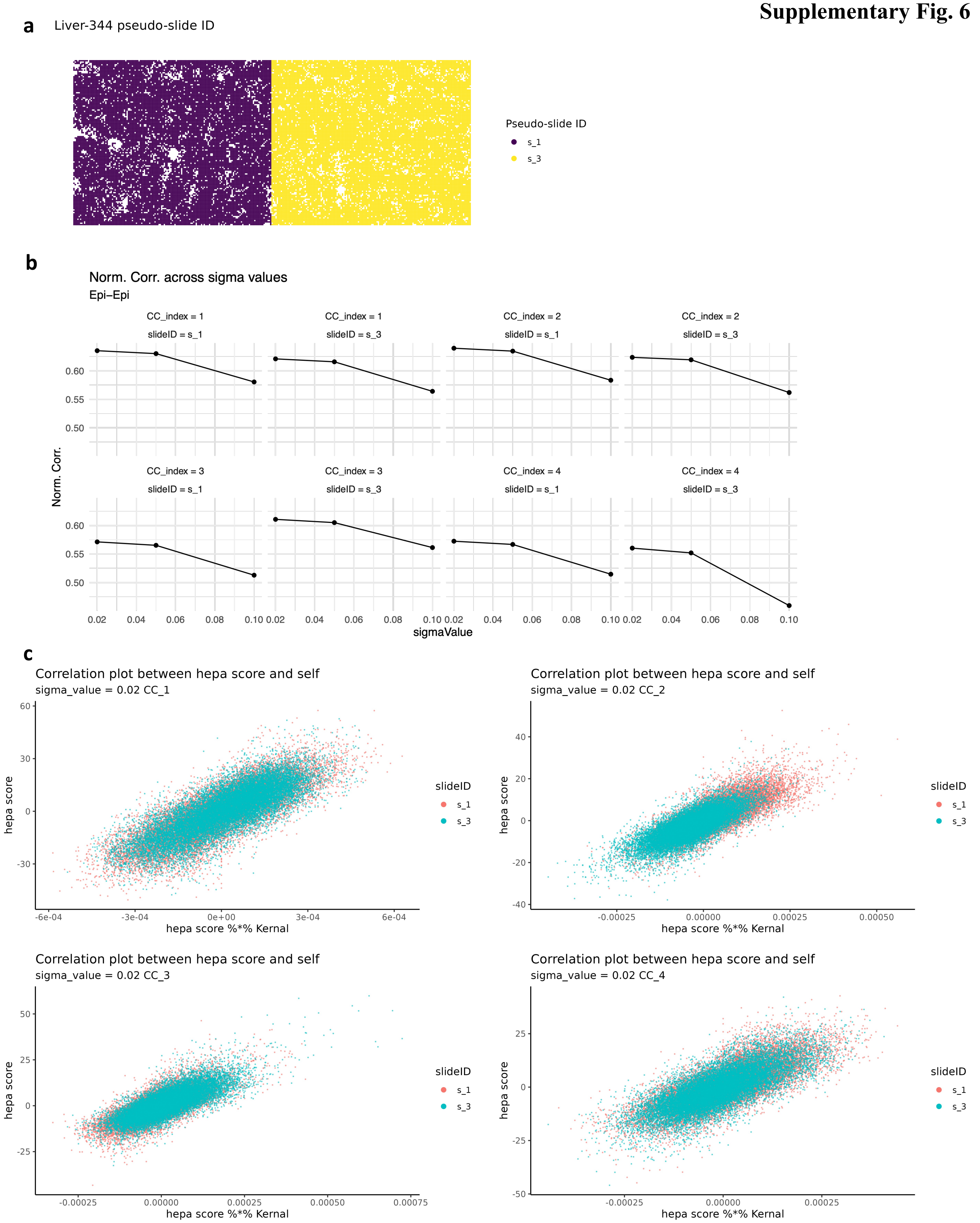

Supplementary Figure 6.

(**a**) Pseudo-slide partition of the Liver-344 data that enables multi-slide CoPro analysis.

(**b**) Normalized correlation across sigma values for Liver-344 hepatocyte CoPro analysis, shown for all four canonical components (CC1–CC4) across all pseudo-slides.

(**c**) Scatter plots of kernel-smoothed hepatocyte scores between pseudo-slide pairs for each of the four canonical components (CC1–CC4) at sigma = 0.02. In each panel, points are colored by pseudo-slide identity (s_1, s_3).

Female Mutant Sample 1
Spatial CoPro Scores

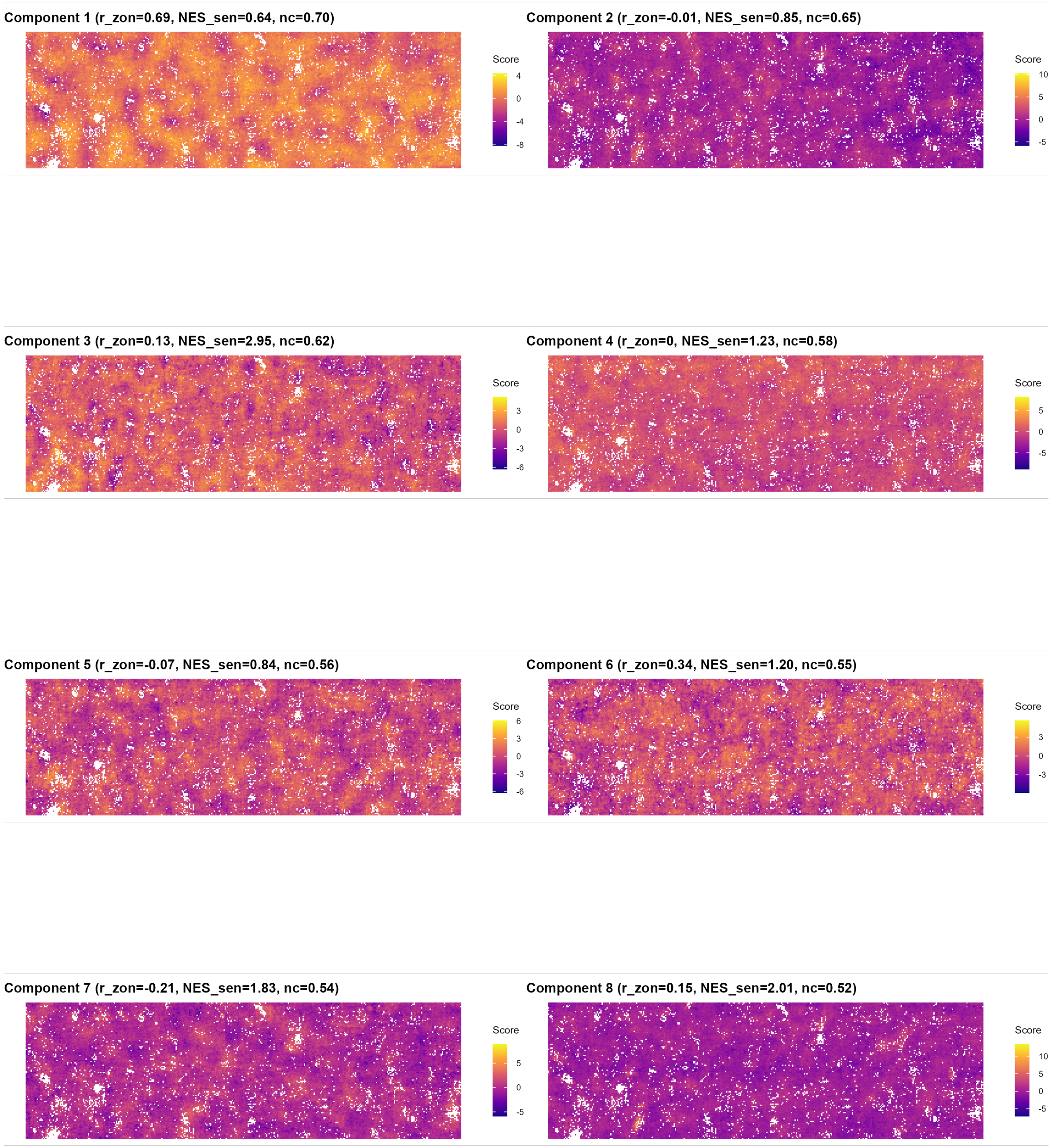

Supplementary Figure 7-12.
Spatial raster plots showing the cell score for each of the eight CoPro components identified in each of the mutant liver samples (female replicates 1-3, followed by male replicates 1-3) Each panel displays the spatial distribution of component scores across the tissue, with color indicating relative enrichment. Component titles indicate the correlation with zonation (r_zon), enrichment for the senescence gene set (NES_sen), and normalized component strength (nc). White regions correspond to vascular structures where no expression measurements were obtained.

Female Mutant Sample 2
Spatial CoPro Scores

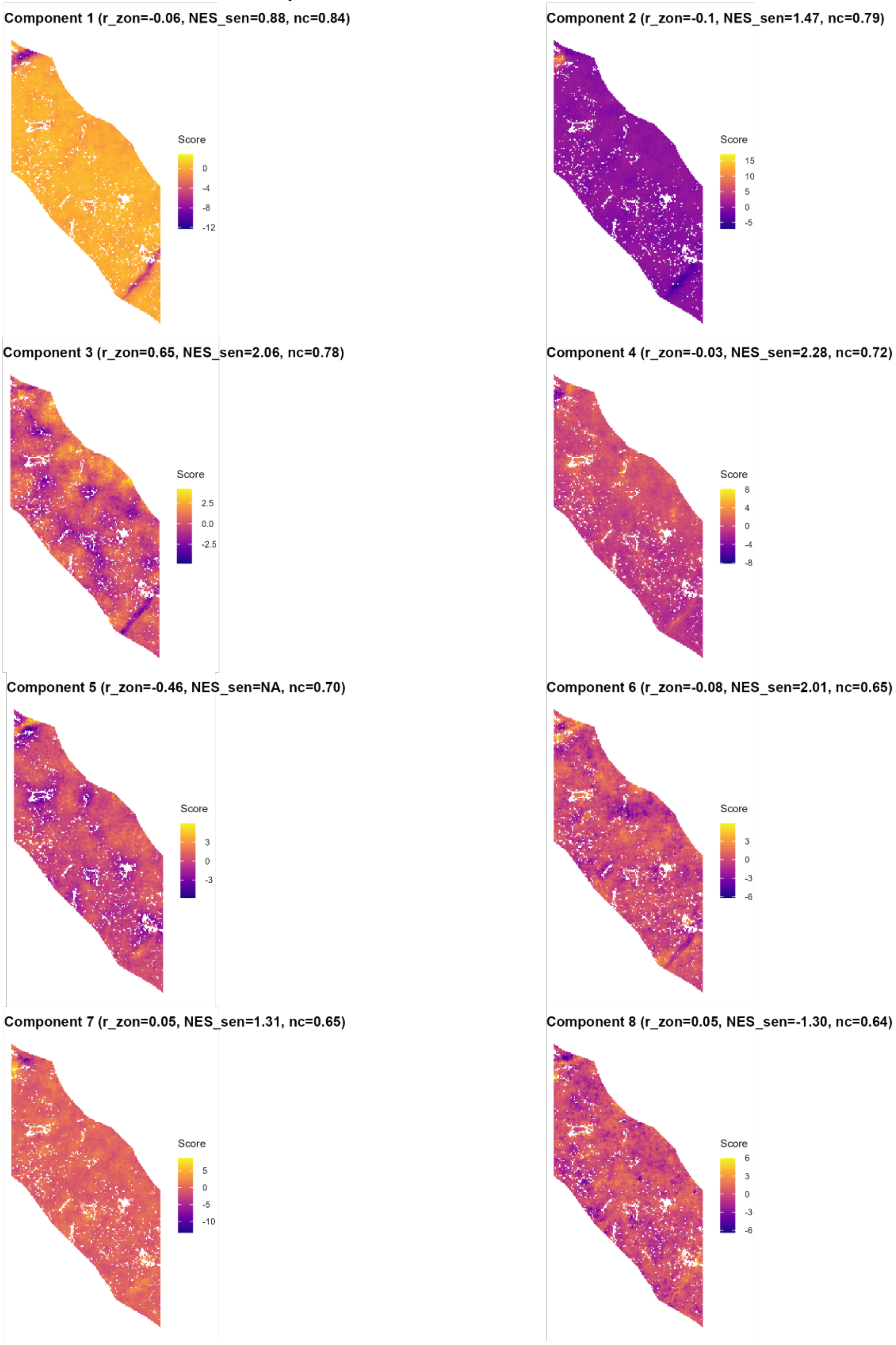

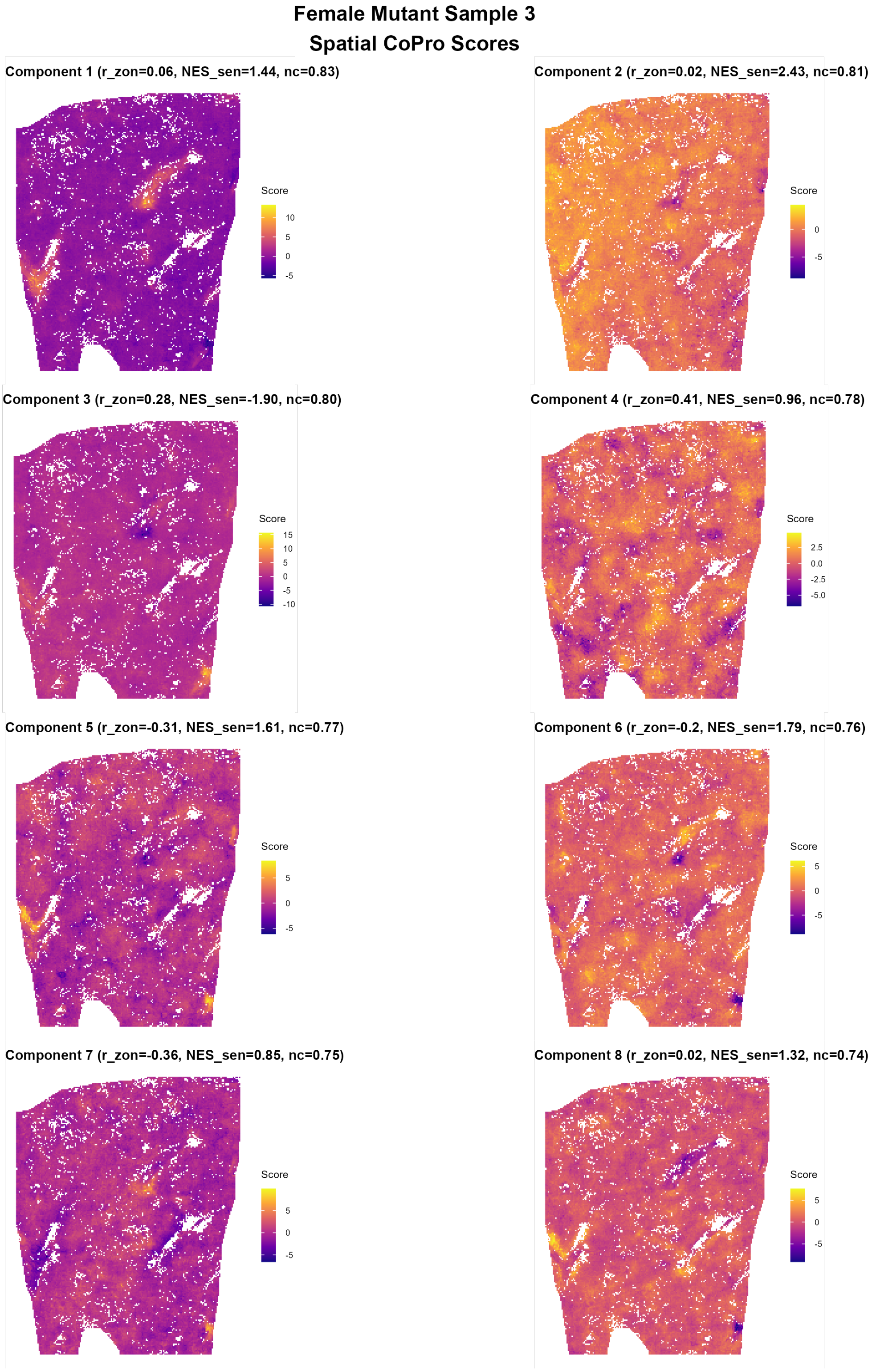
Female Mutant Sample 3
Spatial CoPro Scores
Component 1 (r_zon=0.06, NES_sen=1.44, nc=0.83)
Score
10
5
0
-5
Component 2 (r_zon=0.02, NES_sen=2.43, nc=0.81)
Score
0
-5
Component 3 (r_zon=0.28, NES_sen=-1.90, nc=0.80)
Score
15
10
5
0
-5
-10
Component 4 (r_zon=0.41, NES_sen=0.96, nc=0.78)
Score
2.5
0.0
-2.5
-5.0
Component 5 (r_zon=-0.31, NES_sen=1.61, nc=0.77)
Score
5
0
-5
Component 6 (r_zon=-0.2, NES_sen=1.79, nc=0.76)
Score
5
0
-5
Component 7 (r_zon=-0.36, NES_sen=0.85, nc=0.75)
Score
5
0
-5
Component 8 (r_zon=0.02, NES_sen=1.32, nc=0.74)
Score
5
0
-5

Male Mutant Sample 1
Spatial CoPro Scores

Component 1 (r_zon=0.13, NES_sen=NA, nc=0.82)

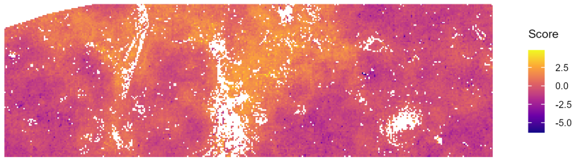

Component 2 (r_zon=-0.25, NES_sen=1.14, nc=0.76)

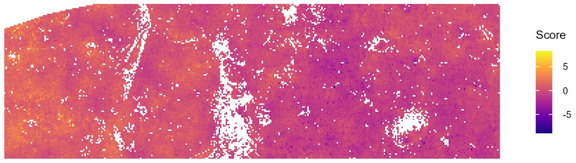

Component 3 (r_zon=0.83, NES_sen=0.76, nc=0.71)

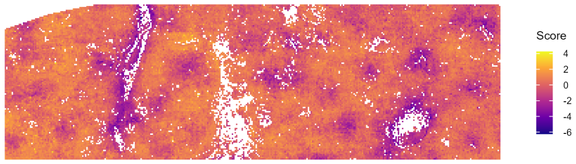

Component 4 (r_zon=0.15, NES_sen=1.24, nc=0.65)

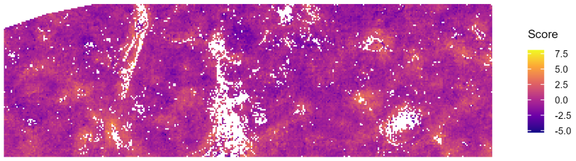

Component 5 (r_zon=-0.12, NES_sen=1.19, nc=0.63)

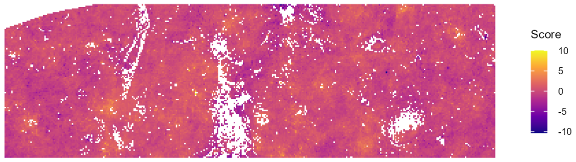

Component 6 (r_zon=0.14, NES_sen=1.51, nc=0.61)

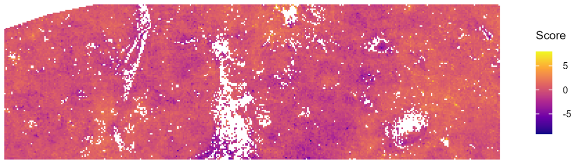

Component 7 (r_zon=0.04, NES_sen=2.75, nc=0.59)

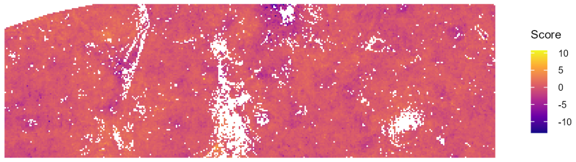

Component 8 (r_zon=-0.16, NES_sen=1.12, nc=0.57)

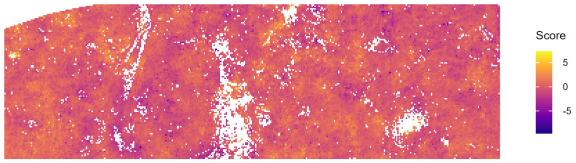

Male Mutant Sample 2
Spatial CoPro Scores

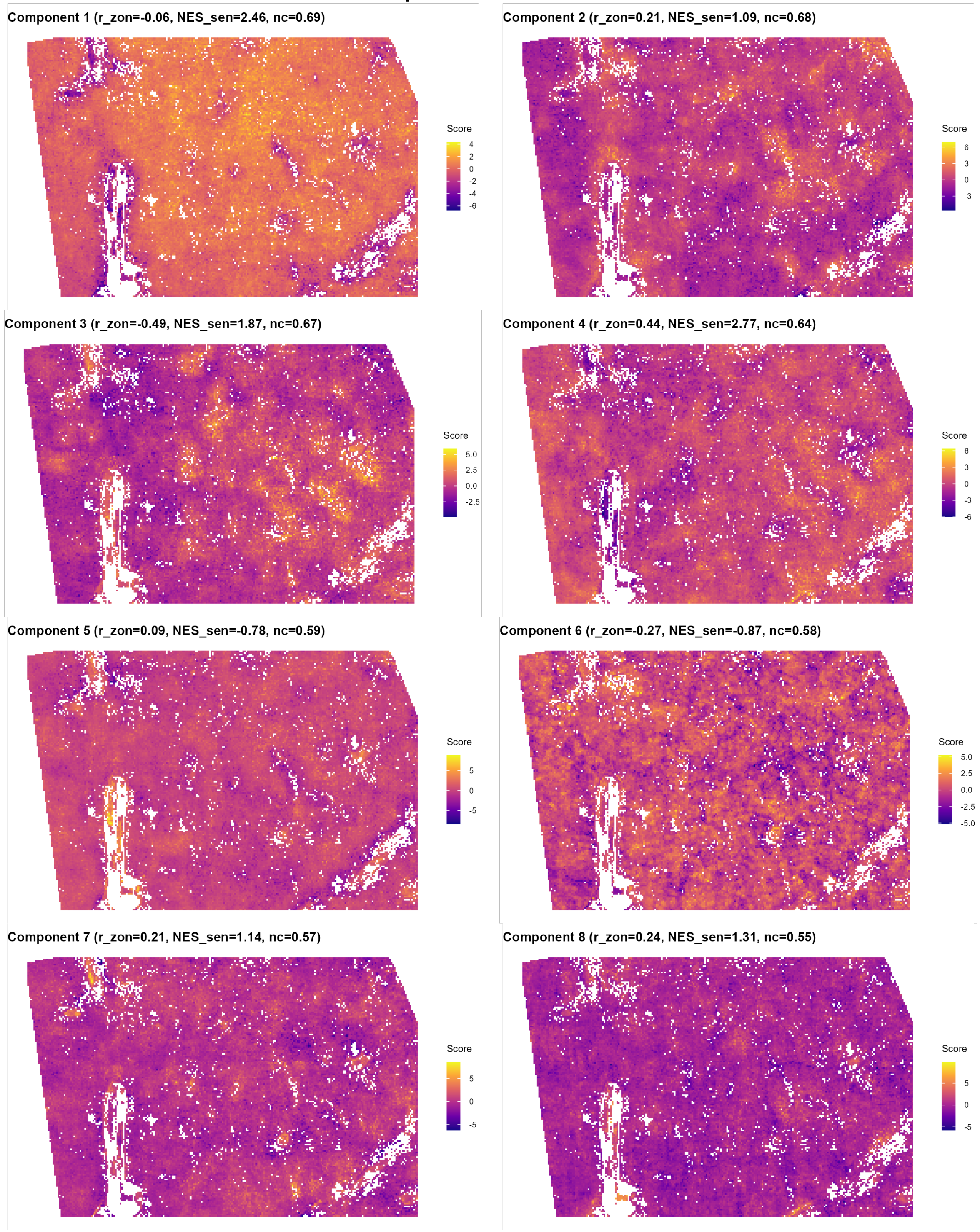

Male Mutant Sample 3
Spatial CoPro Scores

Component 1 (r_zon=-0.36, NES_sen=1.04, nc=0.83)

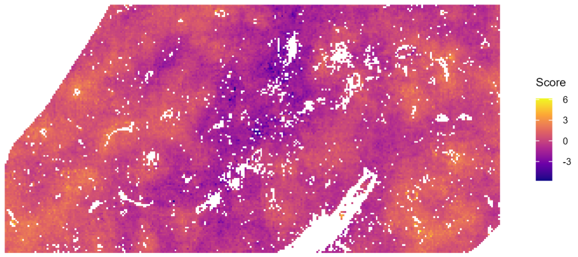

Component 2 (r_zon=-0.69, NES_sen=2.18, nc=0.78)

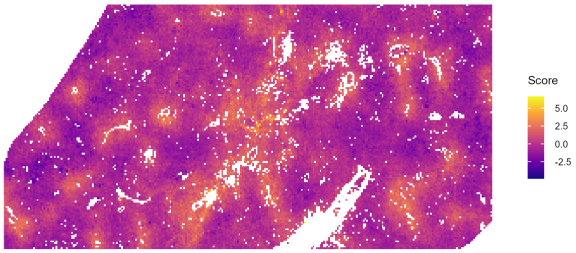

Component 3 (r_zon=-0.09, NES_sen=0.89, nc=0.77)

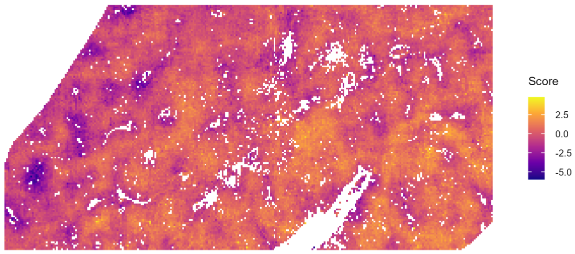

Component 4 (r_zon=-0.06, NES_sen=0.46, nc=0.73)

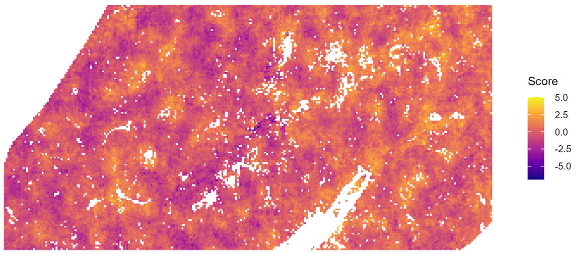

Component 5 (r_zon=0.24, NES_sen=1.61, nc=0.71)

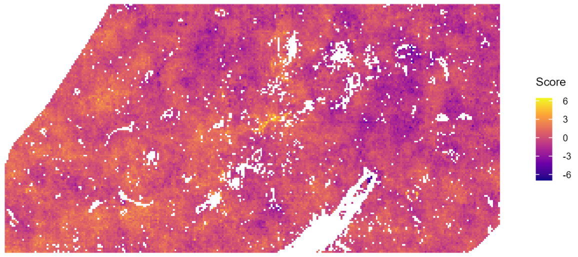

Component 6 (r_zon=0.06, NES_sen=2.53, nc=0.69)

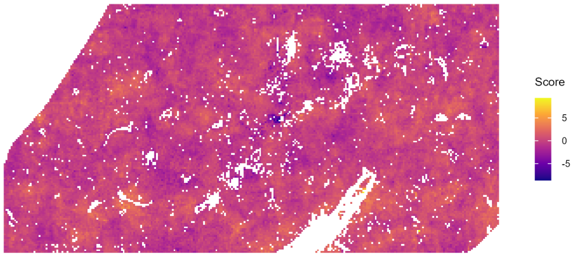

Component 7 (r_zon=0.21, NES_sen=1.11, nc=0.67)

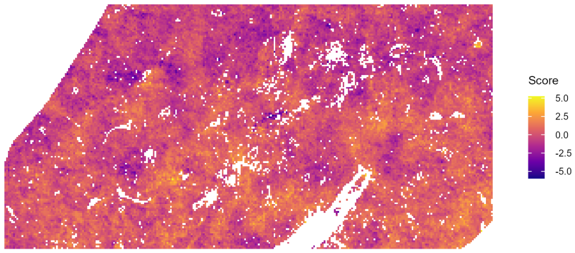

Component 8 (r_zon=0.07, NES_sen=-1.36, nc=0.63)

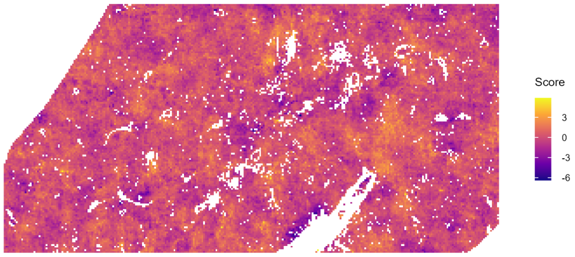

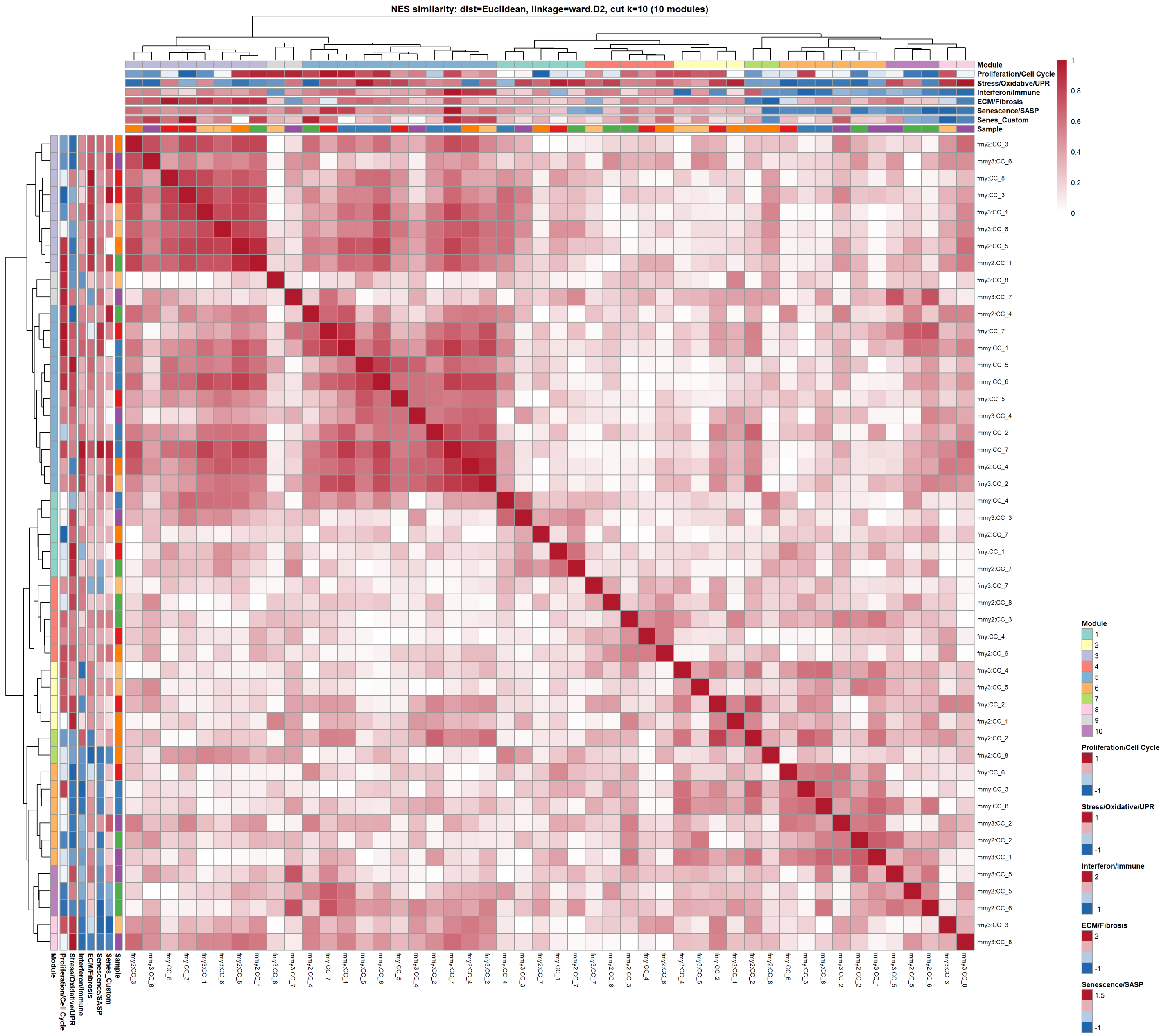

Supplementary Figure 13.
Heatmap showing pairwise similarity between all 48 CoPro components (six samples × eight components per sample), computed from Euclidean distances of normalized pathway enrichment scores (NES) and clustered using Ward.D2 linkage. Components with similar pathway signatures cluster into transcriptional modules (color annotation). Additional annotations summarize enrichment across pathway themes, including proliferation/cell cycle, stress/oxidative response, interferon/immune signaling, extracellular matrix (ECM)/fibrosis, and senescence/SASP programs. This analysis reveals reproducible aging modules shared across animals despite variation in their spatial localization.

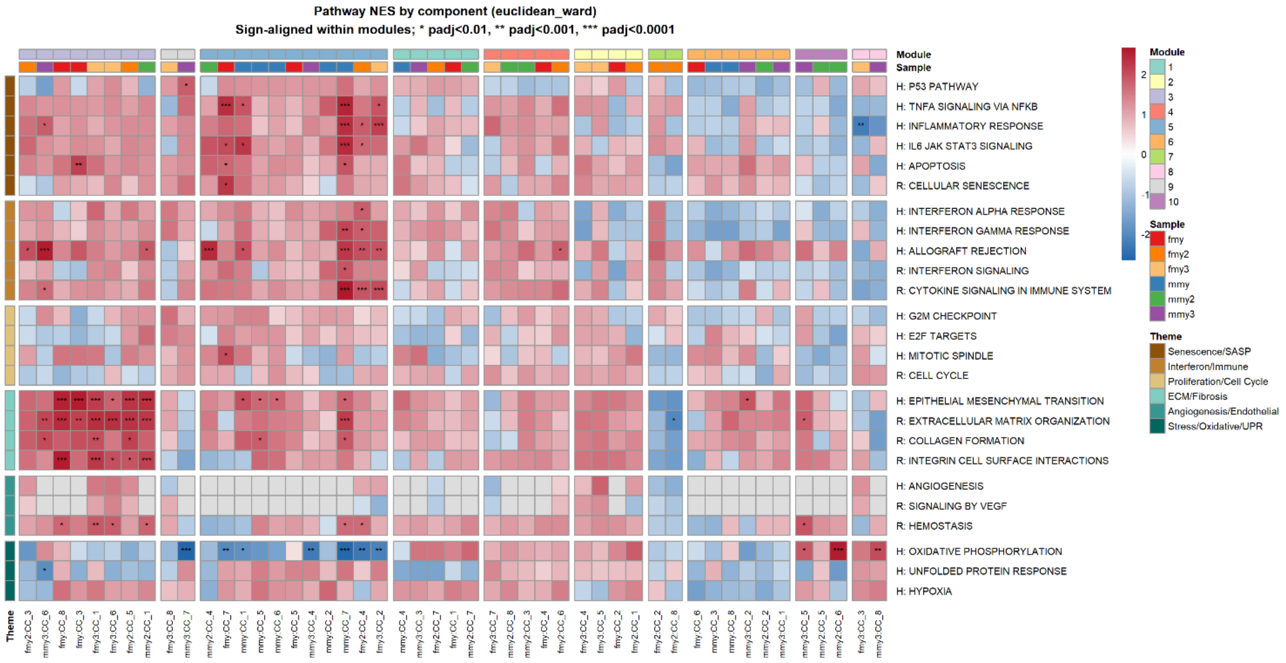

Supplementary Figure 14.

Heatmap showing normalized enrichment scores (NES) for pathway signatures associated with each CoPro component (six samples × eight components per sample). Columns correspond to components and rows to pathways grouped into biological themes including senescence/SASP, interferon/immune signaling, proliferation/cell cycle, ECM/fibrosis, angiogenesis/endothelial programs, and stress/oxidative responses. NES values were sign-aligned within modules to facilitate comparison across components. Components are grouped by hierarchical clustering based on Euclidean distance of pathway enrichment profiles, revealing reproducible transcriptional modules shared across animals. Asterisks indicate statistical significance of pathway enrichment.

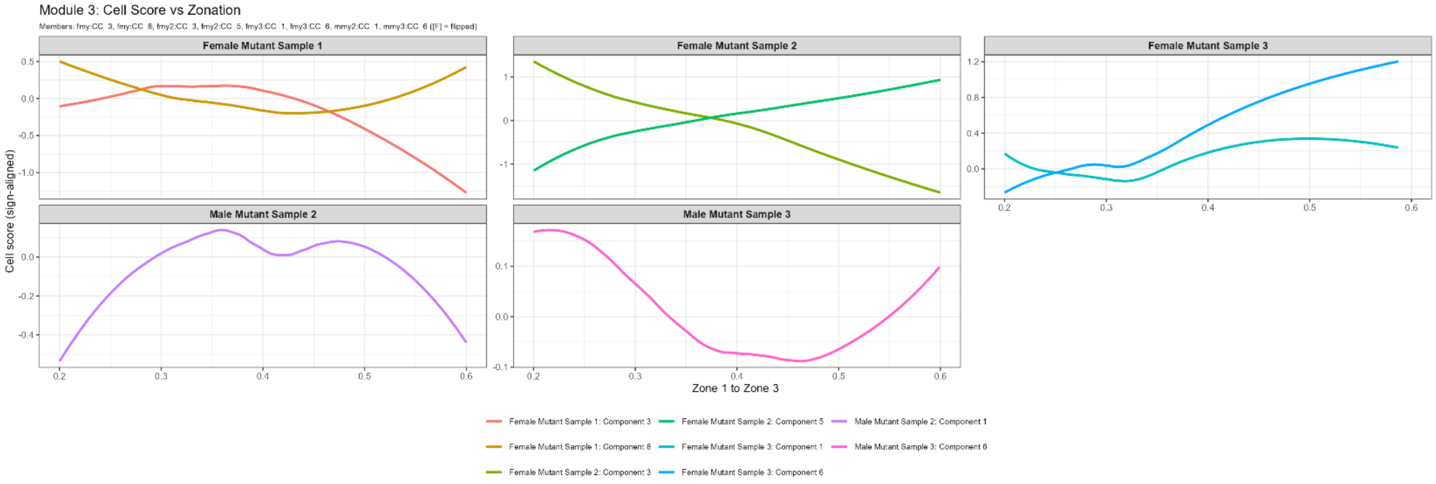

Supplementary Figure 15-17. **Relationship between Module component scores and lobular zonation for modules 3, 5, 6.**

Local spline-fits showing the relationship between CoPro component scores and the zonation coordinate for all components assigned to Module 3 across mutant liver samples. Each curve represents a component from an individual sample, with component scores sign-aligned to enable comparison.

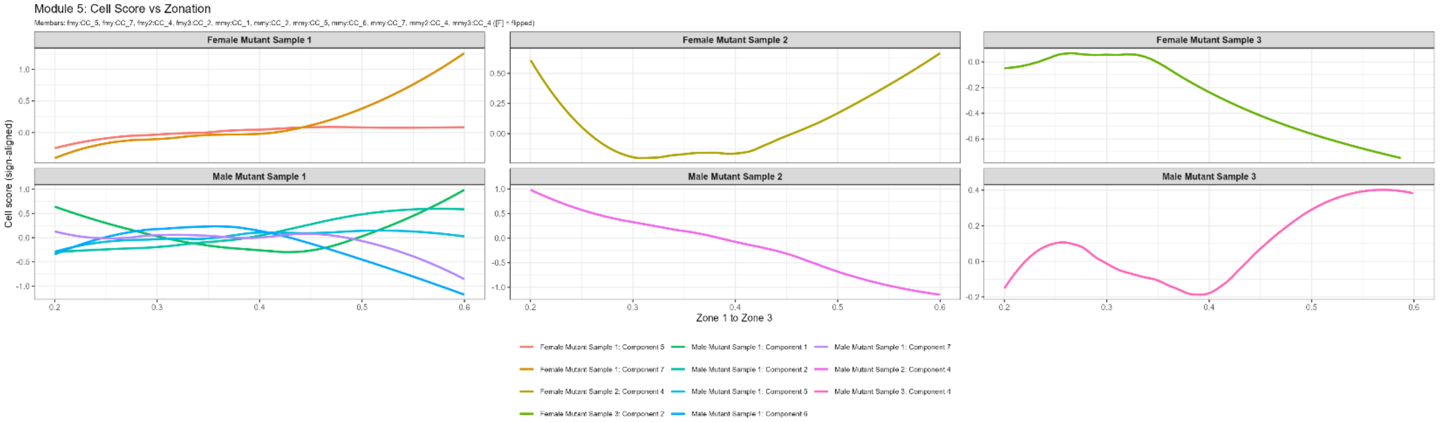

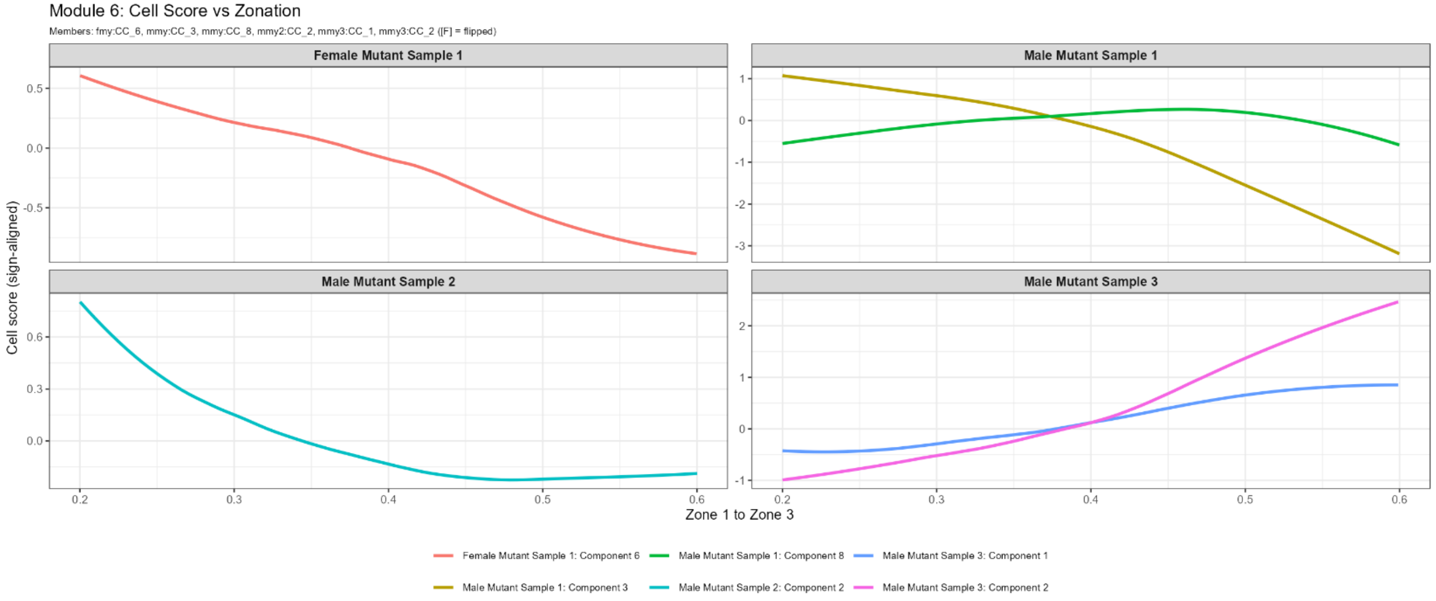

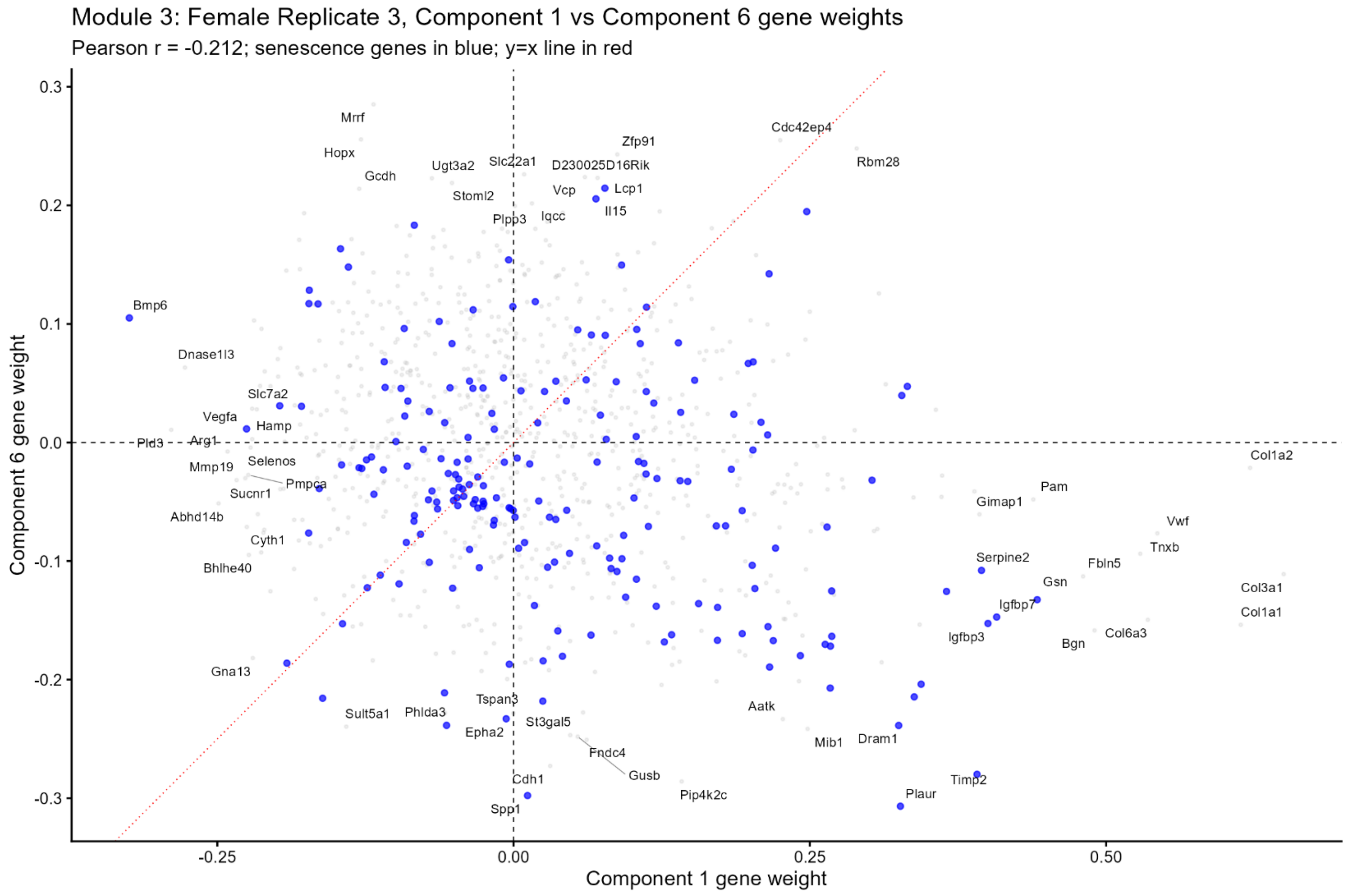

Supplementary Figure 18.
Gene weight comparison of two components within the same ECM-dominated module in female replicate 3. Scatter plot of gene weights for Component 1 (x-axis) versus Component 6 (y-axis), both members of Module 3 in the Euclidean-ward clustering of pathway enrichment profiles. Each point represents one gene; senescence-associated genes are highlighted in blue. The diagonal red line indicates y = x.

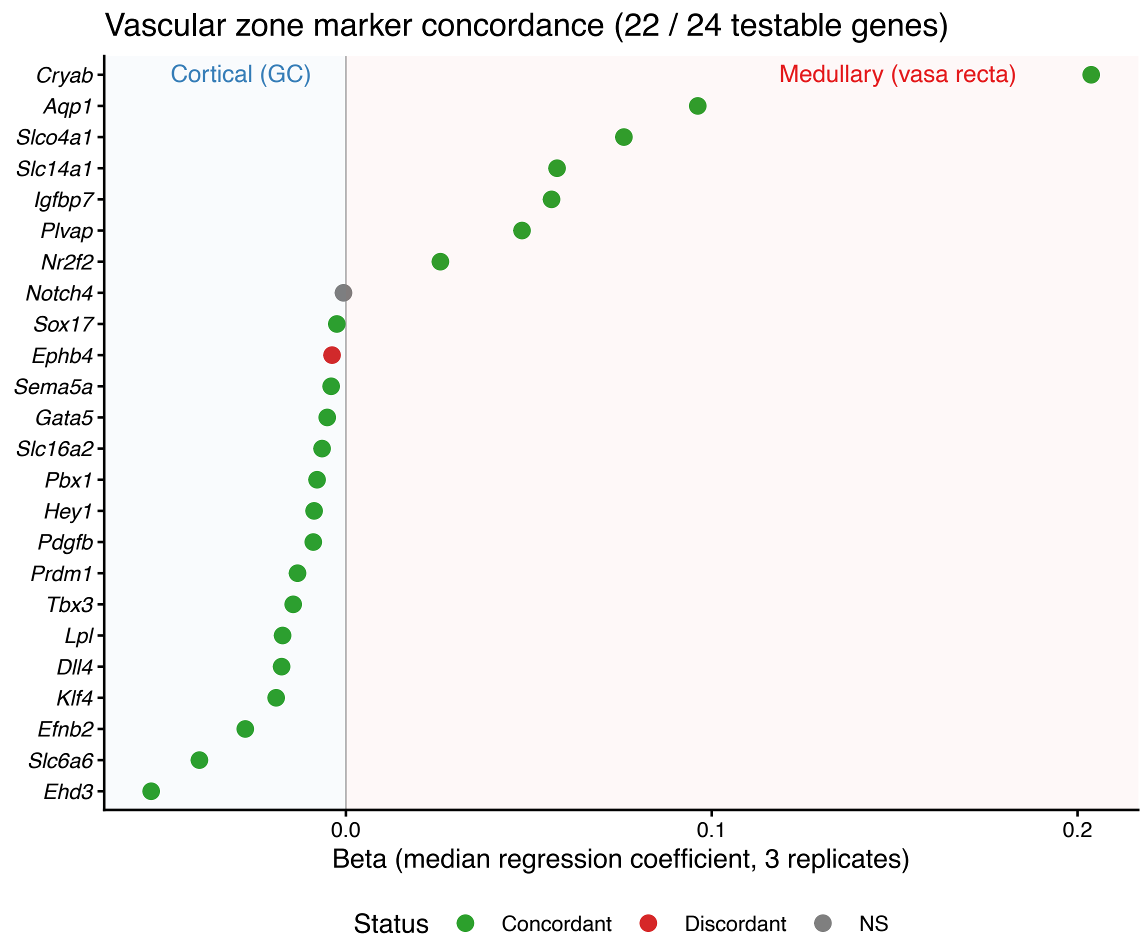

Supplementary Figure 19.
Thirty zone-specific vascular marker genes from Barry et al. (2019) were evaluated against the CoPro-derived corticomedullary vascular axis. Pan-endothelial and non-zone-specific genes were excluded, leaving 24 testable genes (6 were filtered due to expression in <1% of cells). Each gene is plotted by its median regression coefficient (beta) across three replicates, ordered from cortical/glomerular capillary (negative) to medullary/vasa recta (positive). Points are colored by concordance status: concordant (green), discordant (red), or non-significant (grey).
